# Sensitivity to gene dosage and gene expression affects genes with copy number variants observed among neuropsychiatric diseases

**DOI:** 10.1186/s12920-020-0699-9

**Published:** 2020-03-29

**Authors:** Maria Yamasaki, Takashi Makino, Seik-Soon Khor, Hiromi Toyoda, Taku Miyagawa, Xiaoxi Liu, Hitoshi Kuwabara, Yukiko Kano, Takafumi Shimada, Toshiro Sugiyama, Hisami Nishida, Nagisa Sugaya, Mamoru Tochigi, Takeshi Otowa, Yuji Okazaki, Hisanobu Kaiya, Yoshiya Kawamura, Akinori Miyashita, Ryozo Kuwano, Kiyoto Kasai, Hisashi Tanii, Tsukasa Sasaki, Makoto Honda, Katsushi Tokunaga

**Affiliations:** 1 Department of Health Data Science Research, Healthy Aging Innovation Center, Tokyo Metropolitan Geriatric Medical Center, Tokyo, Japan; 2 Laboratory of Evolutionary Genomics, Graduate School of Life Sciences, Tohoku University, Sendai, Japan; 3 Genome Medical Science Project (Toyama), National Center for for Global Health and Medicine, Tokyo, Japan; 4 Department of Human Genetics, Graduate School of Medicine, The University of Tokyo, Tokyo, Japan; 5 Department of Psychiatry and Behavioral Sciences, Tokyo Metropolitan Institute of Medical Science, Tokyo, Japan; 6 RIKEN Center for Integrative Medical Sciences, Kanagawa, Japan; 7 Department of Psychiatry, Hamamatsu University School of Medicine, Hamamatsu, Japan; 8 Department of Child and Adolescent Psychiatry, Hamamatsu University School of Medicine, Shizuoka, Japan; 9 Department of Child Psychiatry, Graduate School of Medicine, The University of Tokyo, Tokyo, Japan; 10 Division for Counseling and Support, The University of Tokyo, Tokyo, Japan; 11 Asunaro Hospital for Child and Adolescent Psychiatry, Mie, Japan; 12 Unit of Public Health and Preventive Medicine, School of Medicine, Yokohama City University, Kanagawa, Japan; 13 Department of Neuropsychiatry, Teikyo University Hospital, Tokyo, Japan; 14 Department of Neuropsychiatry, NTT Medical Center Tokyo, Tokyo, Japan; 15 Department of Psychiatry, Koseikai Michinoo Hospital, Nagasaki, Japan; 16 Panic Disorder Research Center, Warakukai Med Corp, Tokyo, Japan; 17 Department of Psychiatry, Shonan Kamakura General Hospital, Kanagawa, Japan; 18 Department of Molecular Genetics, Bioresource Science Branch, Center for Bioresources, Brain Research Institute, Niigata University, Niigata, Japan; 19 Asahigawaso Research Institute, Asahigawaso Medical-Welfare Center, Okayama, Japan; 20 Department of Neuropsychiatry, Graduate School of Medicine, The University of Tokyo, Tokyo, Japan; 21 Center for Physical and Mental Health, Mie University, Tsu, Mie Japan; 22 Division of Physical and Health Education, Graduate School of Education, The University of Tokyo, Tokyo, Japan

**Keywords:** Copy number variants, Ohnolog, Two-round whole-genome duplication, Gene dosage sensitivity, Gene expression sensitivity, Neuropsychiatric diseases

## Abstract

**Background:**

Copy number variants (CNVs) have been reported to be associated with diseases, traits, and evolution. However, it is hard to determine which gene should have priority as a target for further functional experiments if a CNV is rare or a singleton. In this study, we attempted to overcome this issue by using two approaches: by assessing the influences of gene dosage sensitivity and gene expression sensitivity. Dosage sensitive genes derived from two-round whole-genome duplication in previous studies. In addition, we proposed a cross-sectional omics approach that utilizes open data from GTEx to assess the effect of whole-genome CNVs on gene expression.

**Methods:**

Affymetrix Genome-Wide SNP Array 6.0 was used to detect CNVs by PennCNV and CNV Workshop. After quality controls for population stratification, family relationship and CNV detection, 287 patients with narcolepsy, 133 patients with essential hypersomnia, 380 patients with panic disorders, 164 patients with autism, 784 patients with Alzheimer disease and 1280 healthy individuals remained for the enrichment analysis.

**Results:**

Overall, significant enrichment of dosage sensitive genes was found across patients with narcolepsy, panic disorders and autism. Particularly, significant enrichment of dosage-sensitive genes in duplications was observed across all diseases except for Alzheimer disease. For deletions, less or no enrichment of dosage-sensitive genes with deletions was seen in the patients when compared to the healthy individuals. Interestingly, significant enrichments of genes with expression sensitivity in brain were observed in patients with panic disorder and autism. While duplications presented a higher burden, deletions did not cause significant differences when compared to the healthy individuals. When we assess the effect of sensitivity to genome dosage and gene expression at the same time, the highest ratio of enrichment was observed in the group including dosage-sensitive genes and genes with expression sensitivity only in brain. In addition, shared CNV regions among the five neuropsychiatric diseases were also investigated.

**Conclusions:**

This study contributed the evidence that dosage-sensitive genes are associated with CNVs among neuropsychiatric diseases. In addition, we utilized open data from GTEx to assess the effect of whole-genome CNVs on gene expression. We also investigated shared CNV region among neuropsychiatric diseases.

## Background

Copy number variants (CNVs) have been reported to be associated with diseases, traits, and evolution [1–5]. With new technologies for detecting CNVs, research in these fields have progressed rapidly [6–8]. However, when it comes to clinical applications, several issues still remain. One is that although rare CNVs have been reported to be associated with diseases [2, 3], the rarity of the CNVs makes them difficult to study for elucidating the pathogenicity of the diseases. This is similar to the situation of whole- or exome-sequencing analysis, from which a large number of rare mutations or singletons have been discovered [9]. Secondly, CNVs often span a few megabases and cover several genes [2, 3]; this makes it hard to determine which gene should have priority as a target for further functional experiments. Recent mega biobank projects [10–12] and international data-sharing consortia [13] have enabled the first issue to be overcome; however, the second issue remains unresolved. In this study, we attempted to overcome the second issue by using two approaches: by assessing the influences of gene dosage sensitivity and gene expression sensitivity.

**Fig. 1:**
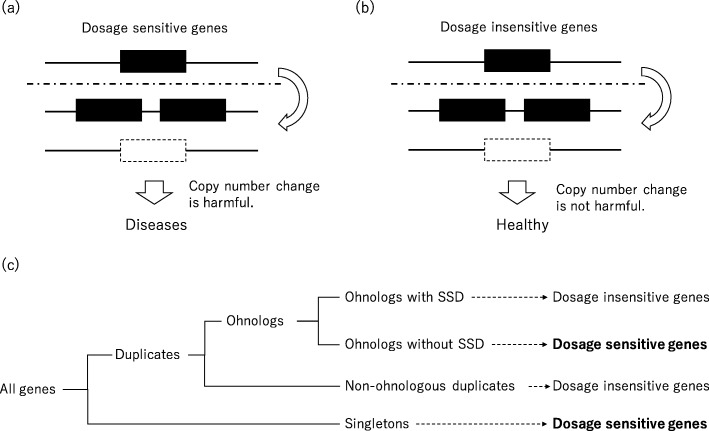
The concept of dosage-sensitive genes and their definitions according to a previous study [17] and our pilot result in S6 Fig. (a) Copy number alterations in dosage-sensitive genes are harmful, but (b) copy number alterations in dosage-insensitive genes are not harmful. (c) According to a previous report [17], all genes can be classified into four groups: ohnologs with small-scale duplications (SSD); ohnologs without SSD; non-ohnologous duplicates; and singletons

Gene dosage sensitivity has been of increasing interest because it might provide a clue for elucidating the pathogenicity of diseases. As such, ClinGen Dosage Sensitivity Map (https://www.ncbi.nlm.nih.gov/projects/dbvar/clingen/) has started to accumulate data on dosage-sensitive genes. The notion of gene dosage sensitivity derived from the gene balance hypothesis, which was suggested in earlier studies [14–16]. In short, the gene balance hypothesis states that a stoichiometric balance is maintained among all of the complex gene products in a pathway, so a copy number change in a single gene of a pathway would be deleterious. Thus, genes under this hypothesis are thought to be dosage-sensitive genes. Copy number alterations, for example, CNVs, in dosage-sensitive genes are harmful and might affect the onset of diseases (Fig. 1(a) and (b)). In contrast, copy number alterations in dosage-insensitive genes are not harmful and might have no effect on the onset of diseases. As such, dosage-sensitive genes may provide useful information in the research of diseases.

Recently, dosage-sensitive genes were effectively identified based on a particular hypothesis and applied to narrow down susceptible genes. In 1970, Susumu Ohno proposed a hypothesis that over the course of evolution, vertebrates experienced genome-wide duplication twice; this is otherwise known as two-round whole-genome duplication (2R-WGD) [18]. Genes that existed at the age of 2R-WGD are called ohnologs, named in honor of Ohno. Studies of ohnologs revealed that the pattern of retained ohnologs was not random and that the currently existing ohnologs are in gene balance as dosage-sensitive genes (Fig. 1(c)) [17, 19]. Several studies have demonstrated the importance and applicability of the dosage sensitivity of ohnologs using CNVs from databases or previously reported pathogenic CNVs [20–23]. Firstly, the susceptibility region for Down syndrome on 21q22.13 was reported to be enriched with ohnologs [19]. Human monogenic disease genes in the Online Mendelian Inheritance in Man (OMIN) (https://www.omim.org) database and previous literature were also found to be enriched in ohnologs [20, 22]. Similarly, previously reported pathogenic genes involved in neuropsychiatric diseases were frequently uncovered to be ohnologs [21, 23].

Previous studies about application of ohnologs limited their survey to target CNVs from databases or previously reported pathogenic CNVs. They did not assess the influence of dosage-sensitive ohnologs on small CNVs or the CNVs detected in each patient. In this study, CNVs > 100 kb in size that were observed in individuals with neuropsychiatric diseases were investigated to assess the burden of the dosage-sensitive ohnologs on these diseases.

**Fig. 2:**
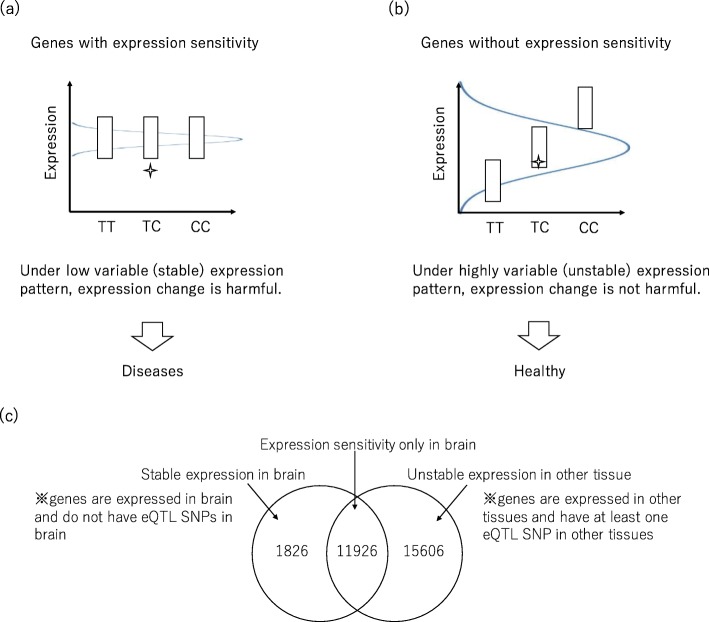
The concept of genes with expression sensitivity only in brain and their definitions, proposed in this study. (**a**) Expression alterations in genes with expression sensitivity are deleterious, while (**b**) expression alterations in genes without expression sensitivity are not deleterious. (c) Genes with expression sensitivity only in brain were defined using GTEx as genes that have stable or low variable expression in brain and unstable or high variable expression in other tissues

Expression quantitative trait locus (eQTL) analysis has been the focus of attention because it allows the assessment of whether single nucleotide polymorphisms (SNPs) or variants affect the expression of genes [24]. However, eQTL analysis does not examine whether an alteration in the gene expression level is deleterious or not. We propose a concept for genes with expression sensitivity (Fig. 2(a) and (b)): modification of the expression level of genes with expression sensitivity is deleterious, while modification of the expression level of genes without expression sensitivity is not deleterious. CNVs are one possible cause of expression level changes because CNVs themselves can change the gene dosage, so our assumption is that CNVs in genes with expression sensitivity might be deleterious. We utilized open data from the Genotype-Tissue Expression (GTEx) project (https://www.gtexportal.org/home/datasets) [24] in order to simulate this concept. Genes with expression sensitivity and, in other words, stable expression in a certain tissue were taken to be genes that are expressed in the tissue and that do not have any eQTL SNPs in the tissue. In contrast, genes without expression sensitivity and, in a different way, unstable expression in a certain tissue were taken to be genes that are expressed in the tissue and that have at least one eQTL SNP in the tissue. Using this definition, we assessed the effect of genes with expression sensitivity in the CNVs observed among neuropsychiatric diseases.

## Methods

### Subjects

The participants in this study were 425 patients with narcolepsy [25], 171 patients with essential hypersomnia (EHS) [26], 595 patients with panic disorders [27], 246 patients with autism [28], 1032 patients with Alzheimer disease [29] and 2135 healthy individuals. All subjects were genotyped in our previous studies, and their data were included for analysis in this study. Ethical approval was obtained from the local institutional review boards of all participating organizations. Age and gender were not matched between the participants with neuropsychiatric diseases and the healthy individuals. All individuals provided written informed consent for their inclusion in this study.

### Genotyping and quality controls

Genomic DNA from all participants was genotyped for 906,622 SNPs using the Affymetrix Genome-Wide SNP Array 6.0 (Thermo Fisher Scientific, Waltham, MA) (S1 Fig). Genotype calling was done using the Birdseed algorithm in Affymetrix Power Tools software (Thermo Fisher Scientific, Waltham, MA). Quality control procedures were performed using PLINK v1.07 (http://zzz.bwh.harvard.edu/plink/). Samples with a call rate < 97% were excluded. For SNP quality control, SNPs with a minor allele frequency < 0.05, a Hardy-Weinberg equilibrium *p* < 0.001 for either the patient group or the healthy control group, and a SNP call rate < 99% were excluded. Samples with a reported family relationship with other participants or a mean probability of being identity-by-descent (PIHAT, calculated in PLINK) value > 0.185 were also excluded. Outliers in the principal component analysis using EIGENSOFT (http://www.hsph.harvard.edu/alkes-price/software/) were also excluded to eliminate population stratification. In the principal component analysis, data from 91 Japanese in Tokyo, Japan (JPT), 90 Han Chinese in Beijing, China (CHB), 180 Utah residents with Northern and Western European ancestry (CEU), and 180 Yoruba in Ibadan, Nigeria (YRI), obtained from the HapMap Project, were also included [30]. Data from the HapMap populations and the present sample sets were combined using common SNPs among all populations after the quality control steps described above.

### CNV detection and quality controls

PennCNV (http://www.openbioinformatics.org/) [31] and CNV Workshop (http://cnv.sourceforge.net) were utilized to detect CNVs (Supporting Information and S1 Fig) [32]. PennCNV employs the Hidden Markov Model. Briefly, PennCNV requires external reference values for the B allele frequency and log R ratio because its algorithm applying the Hidden Markov Model identifies CNV regions using the degree of deviation from these references. We used an in-house reference comprising some of the healthy individuals in our sample set.

After CNV detection by PennCNV, samples with a low detection signal were removed from the subsequent analyses. Samples with a log R ratio standard deviation >|0.3|, B allele frequency drift > 0.01 and CNV call count > 100 were excluded [33, 34]. CNVs with < 10 detection probes and a size < 30 kb were excluded. After the quality controls for population stratification, family relationships and CNV detection, 287 patients with narcolepsy, 133 patients with EHS, 380 patients with panic disorders, 164 patients with autism, 784 patients with Alzheimer disease and 1280 healthy individuals remained.

Gene coordinates were converted from hg18 to hg19 using Liftover (https://genome.ucsc.edu/cgi-bin/hgLiftOver). Artifact regions that tend to cause false-positive CNV detection, centromeric and telomeric regions (±500 kb) and immunoglobulin regions (±500 kb), were removed based on a previous study and software tutorial [3, 31].

### Dosage-sensitive genes

According to a previous study, all genes can be classified into four groups based on evolutionary features and estimation methodology (Fig. 1(c)) [19]. Briefly, genes were divided into singletons and duplicates by means of a BLAST search. Next, among duplicates, genes were classified into ohnologs and non-ohnologous duplicates by comparison with other species. Ohnologs were separated based on whether or not they had undergone small-scale duplication (SSD) after 2R-WGD; these were labelled ohnologs with SSD or ohnologs without SSD. Of these four categories, ohnologs without SSD and singletons were defined as dosage-sensitive genes because genes under the gene balance hypothesis tend not to have undergone SSD after 2R-WGD, and because singletons were found to be less likely to have CNVs in our data (S2 Fig). The classification of human ohnologs was done based on a previous paper by Makino, T et al. using coordinates in Ensembl 73 [19]. According to previous reports, the numbers of dosage-sensitive genes and dosage-insensitive genes are 11,927 and 8360, respectively.

Another classification of ohnologs from OHNOLOGS (http://ohnologs.curie.fr) was utilized by Singh, PP et al. to identify ohnologs [35]. This previous study did not classify ohnologs based on past SSD, so the dosage-sensitive genes were not clearly defined. Here, we used a gene list from OHNOLOGS to validate the results. OHNOLOGS provides results from three different criteria for the identification of ohnologs, and a list of ohnologs defined using the strictest criteria was utilized in our analysis.

### Genes with expression sensitivity *only in brain*

Genes with expression sensitivity *only in brain* were focused on in this study. We made the assumption that if genes with expression sensitivity in any tissue are disrupted, expression balance might be affected not only in brain, but also in other tissues, and it might contribute to neuropsychiatric diseases as well as diseases related to any of the other tissues. However, if genes with expression sensitivity *only in brain* are disrupted, it might only affect expression balance in brain and contribute to neuropsychiatric diseases. Therefore, in this study, genes with expression sensitivity *only in brain* were analyzed to evaluate the genetic background of neuropsychiatric diseases.

Genes with expression sensitivity *only in brain* were defined as those that had stable or low variable expression *in brain* and unstable or high variable expression *in other tissues* (Fig. 2(c)). The GTEx project (https://www.gtexportal.org/home/datasets) [24] was utilized to simulate genes with expression sensitivity in brain. In the database, data from 26 different tissues, including 10 different brain tissues, were registered. (I) (Tissue)_Analysis.v6p.egenes.txt which is a list of genes expressed in each tissue and (II) (Tissue)_Analysis.v6p.signif_snpgene_pairs.txt which is a list of eQTL SNPs within each gene were utilized. Genes with stable expression in a certain tissue were taken to be those that are expressed in that tissue and are thus listed in (I), and those that did not have any eQTL SNPs in the tissue and are thus not listed in (II). Genes with expression sensitivity *in brain* were defined as genes with stable expression in the 10 different tissues from brain. In contrast, genes with unstable expression in a certain tissue were defined as genes that are expressed in that tissue and they are included in (I), and those that have at least one eQTL SNP in the tissue and they are included in (II). Genes with unstable expression *in other tissues* were defined as genes with unstable expression in the 16 different tissues other than brain. Finally, overlapping genes with expression sensitivity *in brain* and unstable expression *in other tissues* were defined as genes with expression sensitivity *only in brain*.

### Statistical analysis

The frequency of CNVs was calculated among the patients of each disease and healthy individuals. CNVs with a size > 100 kb and a frequency < 1% were used in enrichment tests. In the tests, the average number of genes overlapped by CNVs were compared between the cases and the controls for the following categories:(1) dosage-sensitive genes (2); genes with expression sensitivity only in brain; and (3) a combined category of [1, 2]. In more detail, enrichment tests were conducted using the --cnv-count, −-cnv-subset and --cnv-enrichment test options in PLINK to assess whether a subset of genes was enriched relative to all genes.

### Inspection of regions detected only in the patients

**Fig. 3:**
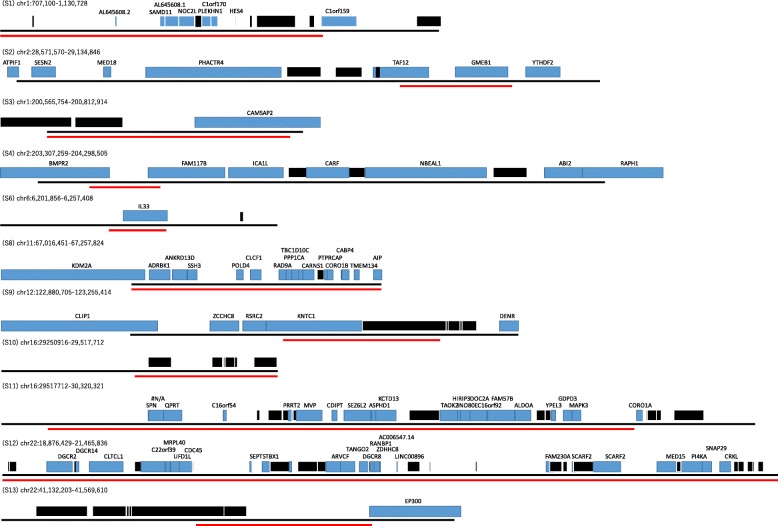
Shared CNV regions and genes among the five neuropsychiatric diseases. Black lines indicate overall regions in which two or three CNVs span. Red lines indicate overlapping regions of CNVs. Blue boxes indicate dosage-sensitive genes with the gene name. Black boxes indicate dosage-insensitive genes without the gene name. Scales are different for each region. Regions S1 to S13 are described in Table 1. Regions S5 and S7 each overlap with only one gene, *CNTNAP2* and *PCDH15*, respectively, so they are not shown in this figure

Regions that were found only in the patients of the five neuropsychiatric diseases were examined. CNVs with a size of > 100 kb and a frequency of < 1% were examined. To narrow down the possible candidate regions, the following criteria were used: (i) previously reported regions; (ii) shared regions among the five neuropsychiatric diseases; or (iii) regions with more than six dosage-sensitive genes. Previously reported regions were taken from the following papers and database: a paper by Itsara, A et al. [36] and a list of candidate genes from the Autism Database (AutDB; http://autism.mindspec.org/autdb/) [37] for autism; a paper by Howe, A et al. [38] for panic disorders; a paper by Lane, J et al. [39] for sleep disorders; a paper by Ripke, S et al. [40] for schizophrenia; and a paper by Van Cauwenberghe, C et al. [41] for Alzheimer disease. Regions with (i) and (ii), or with (ii) and (iii) were listed as shared CNVs among the neuropsychiatric diseases in Table 1 and Fig. 3. The disease-specific regions, defined as those with (iii) and without (i) or with a *p* < 0.05 on Fisher’s exact test, are listed in S7 Table.

**Table 1:**
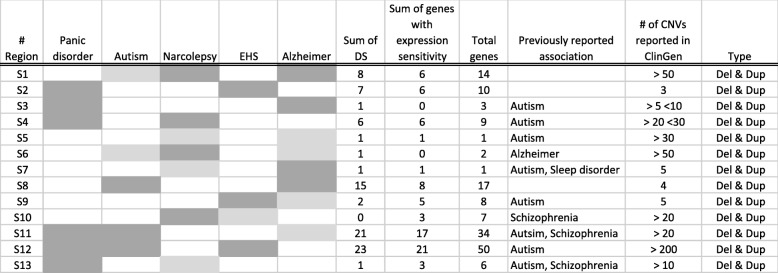
Shared regions among the five neuropsychiatric diseases

Light and dark gray indicate deletion and duplication. Number of dosage sensitive genes, total genes within each region and CNVs reported in ClinGen are shown in this table. Regions S1~13 are used in Fig. 6. Regions S2 and S8 might be novel findings in this study because these regions were not reported previously and but had high density of dosage sensitive genes and few CNV reports in ClinGen

## Results

### Enrichment of dosage-sensitive genes

**Fig. 4:**
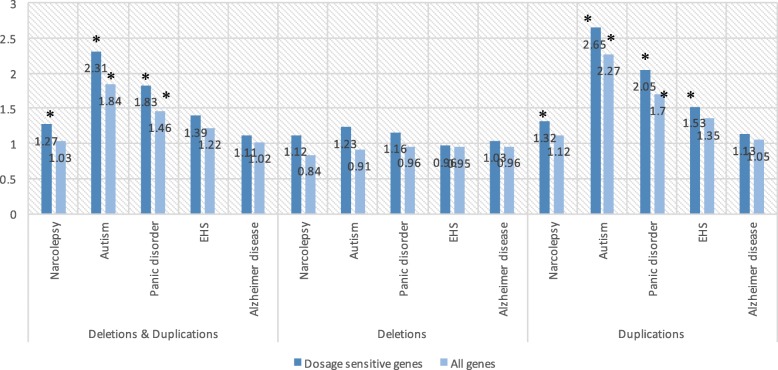
Enrichment of dosage-sensitive genes in CNVs observed in five neuropsychiatric diseases according to the definitions by Makino, T et al. [17]. The y axis shows the relative ratio of the average number of genes spanned by CNVs in comparison to that in healthy controls. CNVs were detected using PennCNV. Asterisks indicate significant enrichment when compared to the healthy individuals in each category

The average number of dosage-sensitive genes was compared between the patients of each disease and the healthy individuals. According to the data from previous reports, 11,927 dosage-sensitive genes and 8360 dosage-insensitive genes were included in the analysis. Overall, in comparison to healthy individuals, a significant enrichment of dosage-sensitive genes was found among individuals with narcolepsy, panic disorders, or autism (Fig. 4 and S1–5 Tables). The similar enrichments in panic disorders and autism were also observed using ohnologs estimated by Singh PP et al. (S3 Fig). In addition, the weaker enrichment was also found using a different CNV detection algorithm (S4 and S5 Figs). In detail, reproducibility of significant enrichments of dosage sensitive genes is partly limited between software, however we could see tendency of enrichment across diseases. Overall, dosage-sensitive genes were significantly enriched in the patients with narcolepsy, panic disorders, or autism. Of note, significant enrichment of dosage-sensitive genes with duplications were observed in all diseases except for Alzheimer disease (Fig. 4). Among the five diseases, patients with panic disorders or autism showed higher enrichment of dosage-sensitive genes with duplications when compared to individuals with narcolepsy or essential hypersomnia (EHS). For deletions, less or no enrichment of dosage-sensitive genes with deletions was seen in the patients when compared to the healthy individuals (Fig. 4).

### Enrichment of genes with expression sensitivity only in brain

**Fig. 5:**
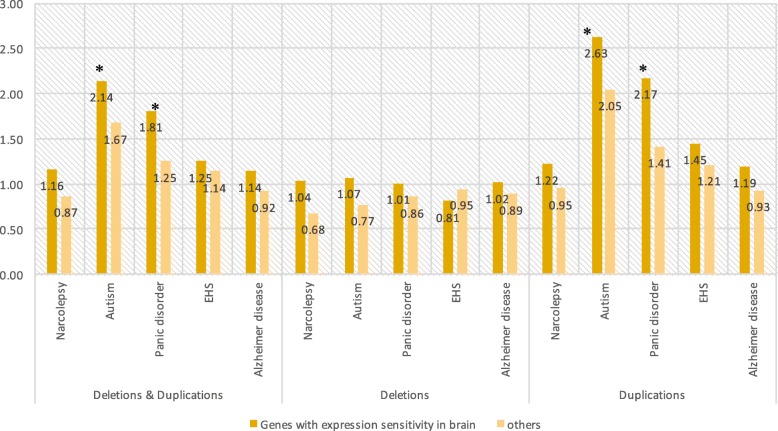
Enrichment of genes with expression sensitivity only in brain in CNVs observed in five neuropsychiatric diseases. The y axis shows the relative ratio of the average number of genes spanned by CNVs in comparison to that in healthy controls. CNVs were detected using PennCNV. Asterisks indicate significant enrichment when compared to the healthy individuals in each category

To demonstrate the effect of CNVs on gene expression, the average number of genes with expression sensitivity only in brain among the CNVs of the five neuropsychiatric diseases were compared between the cases and the controls. In our analysis, we proposed the idea of defining expression sensitivity by utilizing the GTEx database. We defined 11,926 genes as genes with expression sensitivity only in brain from the 39,769 expressed genes in any tissue in the GTEx database (Fig. 2 (c)). Significant enrichment of genes with expression sensitivity only in brain was observed among patients with panic disorders and autism; in contrast, patients with narcolepsy did not show significant enrichment of genes with expression sensitivity only in brain (Fig. 5). While duplications presented a higher burden, deletions did not cause significant differences when compared to the healthy individuals. An enrichment of genes with expression sensitivity only in brain was also seen using a different CNV detection algorithm (S6 Fig). Significant enrichments of genes with expression sensitivity is not partly concordant between software, however we could see slight tendency of enrichment across diseases.

### Combined effect of dosage-sensitive genes and genes with expression sensitivity only in brain

To assess the effect of sensitivity to genome dosage and gene expression at the same time, enrichment tests were also performed. Genes were categorized into four groups based on the combinations of dosage-sensitive genes and genes with expression sensitivity only in brain: (a) dosage-sensitive genes and genes with expression sensitivity only in brain, including 5590 genes; (b) dosage-sensitive genes and genes without expression sensitivity only in brain, including 5527 genes; (c) dosage-insensitive genes and genes with expression sensitivity only in brain, including 3179 genes; and (d) dosage-insensitive genes and genes without expression sensitivity only in brain, including 4480 genes. After converting the genome coordinates to Ensembl 73, 9169 and 10,007 genes were mapped as genes with and without expression sensitivity only in brain, respectively.

**Fig. 6:**
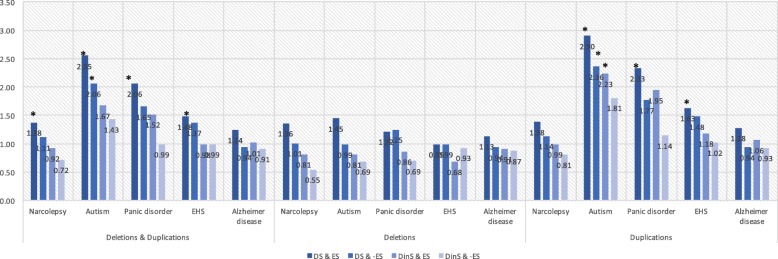
Combined enrichment of dosage-sensitive genes and genes with expression sensitivity only in brain in CNVs observed in five neuropsychiatric diseases in comparison to those in healthy individuals. Human genes were classified into four groups based on the combinations of dosage-sensitive genes and genes with expression sensitivity only in brain: dosage-sensitive genes and genes with expression sensitivity only in brain (DS & ES); dosage-sensitive genes and genes without expression sensitivity only in brain (DS & -ES); dosage-insensitive genes and genes with expression sensitivity only in brain (DinS & ES); and dosage-insensitive genes and genes without expression sensitivity only in brain (DinS & -ES). Asterisks indicate significant enrichment when compared to the healthy individuals in each category

Among the four categories of genes, the group including dosage-sensitive genes and genes with expression sensitivity only in brain showed the highest enrichment among the CNVs from the patients when compared to healthy individuals (Fig. 6). The group including dosage-sensitive genes and genes without expression sensitivity only in brain showed the second highest enrichment, followed by the group including dosage-insensitive genes and genes with expression sensitivity only in brain, then the group including dosage-insensitive genes and genes without expression sensitivity only in brain. A similar tendency was observed in CNVs detected by another software (S6 Table). When the ratio of the average number of genes was compared between cases and controls among all tests for all diseases, the highest ratio of enrichment was observed in the group including dosage-sensitive genes and genes with expression sensitivity only in brain.

### Inspection of regions detected only in the patients

Shared CNV regions among the five neuropsychiatric diseases were also investigated. Table 1 and Fig. 3 show the previously implicated regions or regions with more than six dosage-sensitive genes and regions associated with at least two different diseases. With the use of gene dosage sensitivity, regions S2 (chr1: 28,571,570-29,134,846) and S8 (chr11: 67,016,451-67,257,824) might represent novel findings because these regions have not been reported previously and have few reported CNVs in the ClinGen database (Table 1). In particular, region S8 was significantly enriched in dosage-sensitive genes when compared to the genome-wide ratio of dosage-sensitive genes and dosage-insensitive genes (*p* = 0.013 on Fisher’s exact test), and while region S2 was not significantly enriched (*p* = 0.540 on Fisher’s exact test), it had a higher density than the genome-wide average (density of dosage-sensitive genes at region S2: 70.0%; genome-wide average of dosage-sensitive genes: 58.7%). Disease-specific regions were also examined, including regions with more than six dosage-sensitive genes; regions that have not been reported previously; or regions with a *p* < 0.05 on Fisher’s exact test. All diseases except for narcolepsy had disease-specific regions that fulfilled the above criteria.

## Discussion

In this paper, the phenotypic impact of sensitivity to gene dosage was demonstrated using CNVs detected in patients of five neuropsychiatric disorders. These results were concordant with a previous study using intolerance score [8]. The previous study utilized a ranking system that scored genes according to their tolerance to functional variation and demonstrated that CNVs among patients with schizophrenia had higher genic intolerance scores than that of healthy controls. In other words, CNVs in schizophrenia occurred within genes intolerance to functional variations. This is similar to our results, because it was known that genes with dosage sensitivity hardly experienced copy number alteration by CNV [19].

Duplications appeared to have a substantial impact on disease onset, as we saw from the significant enrichment of dosage-sensitive genes with duplications in all diseases except for Alzheimer disease. Until recently, duplications were often deprioritized and analyzed after deletions, perhaps because duplications are thought to be less harmful than deletions [3]. However, a recent study suggested that duplications exhibit more diversity, weaker selective constraint, and a four-fold greater chance of affecting genes than deletions, indicating that they possess signatures of adaptive evolution [5]. Indeed, several studies have observed that high-copy CNVs were associated with human traits [4, 42–45]. It might be because once deletions occurred, they disappeared quickly or their frequencies were reduced in a population because they were lethal or extremely harmful and disadvantageous. However, when duplications occurred, they were retained within the diversity of a population because they were not lethal nor sufficiently harmful or disadvantageous to have had their frequencies reduced. From the viewpoint of the total number of dosage-sensitive genes affected by CNVs, the accumulative impact of duplications might be larger than that of deletions. This study provides another example that demonstrates the importance of duplications in the onset of diseases.

Genes with sensitive expression only in brain were identified utilizing publically available GTEx data, and the effects of CNVs on the expression of these genes were evaluated. Similar to the gene balance hypothesis, expression balance might also be maintained; thus, alterations in the gene expression level of genes with expression sensitivity are deleterious, while such alterations in genes without expression sensitivity are not. Indeed, it is already reported that dosage change of expressed genes in brain are less frequent than those of other genes and are controlled by tighter transcriptional regulation [46]. Also, according to an analysis using intolerance score, genes highly expressed in the brain showed the most intolerance to CNV [8].CNVs could be one cause of change at the expression level because CNVs modify gene dosage, so it is possible that CNVs in genes with expression sensitivity might contribute to the onset of diseases.

Our finding of no significant enrichment of genes with expression sensitivity only in brain among individuals with narcolepsy suggests that the genetic background of narcolepsy may not be related to brain function, at least not in relation to CNVs. Previous studies of narcolepsy showed an autoimmune etiology for the disease and orexin (hypocretin) deficiency among patients with narcolepsy. HLA-DQB1*06:02 was a major genetic factor for the onset of narcolepsy [47–51], and the T cell receptor alpha gene (*TRA*) and purinergic receptor P2Y, G-protein coupled, 11 gene (*P2RY11*) were also reported to be associated with narcolepsy [52, 53]. Pathway analysis of CNVs in patients with narcolepsy found enrichment of immune-related pathway [54]. Additionally, the hypocretin-1 level was found to be reduced or undetectable in the cerebrospinal fluid of narcoleptic patients [55], and postmortem examination showed a marked reduction of hypocretin-producing neurons in the hypothalamus [56]. Nevertheless, regarding CNVs, our result of no significant enrichment of genes with expression sensitivity only in brain demonstrated that overall, narcolepsy might be an autoimmune disease rather than a brain-related disease.

In this study, we proposed a method of assessing the influence of CNVs on gene expression. Analysis of eQTL enables the evaluation of whether SNPs or variants affect the gene expression level [24]; however, these analysis does not assess whether alterations of the gene expression level are deleterious or not. Here, genes with expression sensitivity were able to evaluate influence of change at expression-level. In addition to expression sensitivity, it is necessary to assess the impact of CNVs on gene expression [57] because CNVs contribute to 18 to 99% of the expression level of genes [58, 59]. Indeed, previous studies analyzed expression-level of CNV locus using human brain tissue from healthy individuals and patients with neuropsychiatric diseasaes [60, 61]. Yet, it has been difficult to know the effect of each CNVs on gene expression easily because there is no gene expression reference panel for CNVs like there is for SNPs. In this study, we proposed a cross-sectional omics approach using publicly available data. To the best of our knowledge, this is a novel method for assessing the influence of CNVs on gene expression.

The shared regions among five neuropsychiatric diseases were assessed. With the use of gene dosage sensitivity, regions S2 and S8 may be novel findings (Table 1 and Fig. 3). Region S8 was shared between autism and Alzheimer disease, and within region S8, the carnosine synthase 1 gene (*CARNS1*) was highly expressed in brain. This gene was known to catalyze the formation of carnosine and homocarnosine. Base on ARCHS4 database, top predicted biological process in Gene Ontology is fatty acid elongation [62]. Increased expression of fatty acid synthesis in model of autism was demonstrated previously [63]. Correlation between deficient biosynthesis of fatty acid and cognitive impairment in Alzheimer’s disease was reported [64]. This gene might be involved pathogenesis of these diseases. The protein tyrosine phosphatase receptor type C-associated protein gene (*PTPRCAP*) was reported to contain one of the top differentially methylated probes in autism [65]. A genetic link between autism and Alzheimer disease has previously been reported, and this provides additional evidence for a shared background between autism and Alzheimer disease [66].

Disease-specific regions of five neuropsychiatric diseases were evaluated. For panic disorders, region P3 spanned seven dosage-sensitive genes (S7 Table). One of them was the solute carrier family 17 member 7 gene (*SLC17A7*); this gene is highly expressed in brain, and it was reported that expression of this gene resulted in the uptake of glutamate as a vesicular glutamate transporter [67]. Reduced expression of this gene leads to reduced uptake of glutamate and an increased amount of glutamate in brain. Previously, a high amount of glutamate was reported to be associated with panic attacks [68]. Therefore, it seems that *SLC17A7* within this deletion may be a candidate causative gene in patients with panic disorders. Region P2 (chr14:50,043,390-50,311,552) spanned eight dosage-sensitive genes. Among these genes, the ribosomal protein S29 gene *(RPS29*) showed a marginal significant association in a genome-wide association study with posttraumatic stress disorder [69]. Another gene, the kelch domain containing 1 gene (*KLHDC1*), was reported to show a moderate significant association with bipolar disease [70]. Four individuals with panic disorders had duplications in region P1 (chr4:39,500,375-39,784,412; *p* = 0.0027 on Fisher’s exact test). The ubiquitin-conjugating enzyme gene (*UBE2K*) and small integral membrane protein 14 gene (*SMIM14*) were overlapped in all duplications among these four individuals. *UBE2K* was reported to be correlated with positive symptoms of psychosis in schizophrenia and bipolar patients [71]. By thoroughly inspecting dosage-sensitive genes within CNVs, it seems possible to narrow down candidate genes.

We found several regions with more than six dosage-sensitive ohnologs in Alzheimer disease. No increase in the average number of CNVs per person was observed in Alzheimer disease. Nevertheless, when we further investigated each CNV that occurred only among patients, Alzheimer disease seemed to associate with dosage-sensitive genes. This result was concordant with the results of a recent paper [23]. In particular, a duplication in region AD6 (chr11: 104,756,445-107,834,208) was observed in one case in this study (S7 Table), and it overlapped with the contactin 5 gene (*CNTN5*) and ELMO domain-containing 1 gene (*ELMOD1*), which were reported as candidate genes in the recent paper. In addition, when we investigated CNVs with a size < 100 kb, the amyloid beta precursor protein gene (*APP*) overlapped with a deletion from one patient with Alzheimer disease in this study. Previously, duplications of *APP* were reported to be causative variants for early onset familial Alzheimer disease [72–74]. Although a deletion was observed in this study, *APP* was a dosage-sensitive gene, so it is possible that not only a gain, but also a loss of gene dosage might have contributed to the onset of disease in this patient. Therefore, both the total burden of CNVs and inspection of dosage-sensitive genes around CNVs are useful strategies for identifying susceptibility genes.

The scope of our study was limited to ohnologs that underwent 2R-WGD and were very ancient. However, recent human-specific segmental duplications are also known to contribute to disease onset or phenotypic diversity [75]. It is important to inspect CNVs from both viewpoints. In addition, we only evaluated the effect of CNVs on neighboring genes. It was reported that among genes with expression influenced by CNVs, 53% of the expression is affected by CNVs that are distant from the target genes [1]. However, our analysis did not consider the distant effects of CNVs. Also the method we proposed in this study simplify expression among brain and did not consider different gene expression of region in brain and dynamics of gene expression through life span so on.

## Conclusions

In this study, we demonstrated the impact of sensitivity to gene dosage and gene expression using CNVs identified in patients with neuropsychiatric diseases. A novel approach was proposed, and the effect of CNVs on gene expression was globally assessed. These results will help elucidate the pathogenicity of diseases more clearly than before.

## Supplementary information

**Additional file 1: Figure S1.** Overview of the study. CNVs identified from each detection method were filtered and analyzed independently. **Figure S2.** CNV frequency and proportion of dosage-sensitive genes overlapped by CNVs. The x axis shows the frequency of CNVs in each patient and healthy individual. The y axis shows the proportion of ohnologs with SSD, singletons, ohnologs without SSD, or non-ohnologous duplicates. **Figure S3.** Enrichment of ohnologs in CNVs observed in five neuropsychiatric diseases according to the definitions by Singh, PP et al. **Figure S4.** Enrichment of dosage-sensitive genes in CNVs observed in five neuropsychiatric diseases with the use of another software, CNV Workshop. **Figure S5.** Enrichment of ohnologs in CNVs observed in five neuropsychiatric diseases with the use of another software, CNV Workshop. **Figure S6.** Enrichment of genes with expression sensitivity only in brain in CNVs observed in five neuropsychiatric diseases with the use of another software, CNV Workshop. **Table S1.** Enrichment of dosage-sensitive ohnologs in individuals with narcolepsy using CNVs detected by PennCNV. **Table S2.** Enrichment of dosage-sensitive ohnologs in individuals with autism using CNVs detected by PennCNV. **Table S3.** Enrichment of dosage-sensitive ohnologs in individuals with panic disorders using CNVs detected by PennCNV. **Table S4.** Enrichment of dosage-sensitive ohnologs in individuals with essential hypersomnia using CNVs detected by PennCNV. **Table S5.** Enrichment of dosage-sensitive ohnologs in individuals with Alzheimer disease using CNVs detected by PennCNV. **Table S6.** Combined enrichment of dosage-sensitive genes and genes with expression sensitivity only in brain in CNVs observed in five neuropsychiatric diseases with the use of another software, CNV Workshop. **Table S7.** Disease-specific regions for five neuropsychiatric diseases.

## Data Availability

The datasets used in the current study are available from National Bioscience Database Center (https://biosciencedbc.jp) and from the authors with the reasonable request.

## Abbreviations

2R-WGD: Two-round whole-genome duplication
APP: Amyloid beta precursor protein gene
AutDB: Autism Database
CARNS1: Carnosine synthase 1 gene
CEU: Utah residents with Northern and Western European ancestry
CHB: Han Chinese in Beijing, China
CNTN5: Contactin 5 gene
CNVs: Copy number variants
EHS: Essential hypersomnia
ELMOD1: ELMO domain-containing 1 gene
EQTL: Expression quantitative trait locus
GTEx: Genotype-Tissue Expression
HLA-DQB1: Major histocompatibility complex, class II, DQ beta 1
JPT: Japanese in Tokyo, Japan
KLHDC1: Kelch domain containing 1 gene
OMIN: Online Mendelian Inheritance in Man
P2RY11: Purinergic receptor P2Y, G-protein coupled, 11 gene
PIHAT: Probability of being identity-by-descent
PTPRCAP: Protein tyrosine phosphatase receptor type C-associated protein gene
RPS29: Ribosomal protein S29 gene
SLC17A7: Solute carrier family 17 member 7 gene
SMIM14: Small integral membrane protein 14 gene
SNPs: Single nucleotide polymorphisms
SSD: Small-scale duplication
TRA: T cell receptor alpha gene
UBE2K: Ubiquitin-conjugating enzyme gene
YRI: Yoruba in Ibadan, Nigeria
