# Supplementary material for: Sensitivity to gene dosage and gene expression affects genes with copy number variants observed among neuropsychiatric diseases
Source: BMC Med Genomics. 2020 Mar 29;13:55. doi: 10.1186/s12920-020-0699-9 (PMC7104509; doi:10.1186/s12920-020-0699-9)
Supplement: Supplementary file 1 — Additional file 1: Figure S1. Overview of the study. CNVs identified from each detection method were filtered and analyzed independently. Figure S2. CNV frequency and proportion of dosage-sensitive genes overlapped by CNVs. The x axis shows the frequency of CNVs in each patient and healthy individual. The y axis shows the proportion of ohnologs with SSD, singletons, ohnologs without SSD, or non-ohnologous duplicates. Figure S3. Enrichment of ohnologs in CNVs observed in five neuropsychiatric diseases according to the definitions by Singh, PP et al. Figure S4. Enrichment of dosage-sensitive genes in CNVs observed in five neuropsychiatric diseases with the use of another software, CNV Workshop. Figure S5. Enrichment of ohnologs in CNVs observed in five neuropsychiatric diseases with the use of another software, CNV Workshop. Figure S6. Enrichment of genes with expression sensitivity only in brain in CNVs observed in five neuropsychiatric diseases with the use of another software, CNV Workshop. Table S1. Enrichment of dosage-sensitive ohnologs in individuals with narcolepsy using CNVs detected by PennCNV. Table S2. Enrichment of dosage-sensitive ohnologs in individuals with autism using CNVs detected by PennCNV. Table S3. Enrichment of dosage-sensitive ohnologs in individuals with panic disorders using CNVs detected by PennCNV. Table S4. Enrichment of dosage-sensitive ohnologs in individuals with essential hypersomnia using CNVs detected by PennCNV. Table S5. Enrichment of dosage-sensitive ohnologs in individuals with Alzheimer disease using CNVs detected by PennCNV. Table S6. Combined enrichment of dosage-sensitive genes and genes with expression sensitivity only in brain in CNVs observed in five neuropsychiatric diseases with the use of another software, CNV Workshop. Table S7. Disease-specific regions for five neuropsychiatric diseases. [file 12920_2020_699_MOESM1_ESM.docx]

**Supporting Information**

**CNV detection and quality controls using another software**

In addition to PennCNV (http://www.openbioinformatics.org/)(1), another software, CNV Workshop (http://cnv.sourceforge.net)(2), was used to improve the reliability of our analysis. CNV Workshop utilizes a circular binary segmentation algorithm. Briefly, CNV Workshop did not require any reference values, and it infers CNVs by internal comparison of the log R ratio with neighboring regions in an individual. The CNVs from each software were analyzed separately, so the results did not depend on the different characteristics of the two software programs or ease of CNV detection at a certain locus.

After CNV detection by CNV Workshop, samples with a log R ratio standard deviation >|0.3|, B allele frequency drift >0.01 and CNV call count >100 were excluded. Finally, 281 patients with narcolepsy, 118 patients with EHS, 387 patients with panic disorders, 165 patients with autism, 785 patients with Alzheimer disease and 1,285 healthy individuals passed the quality controls for population stratification, family relationships and CNV detection.

Gene coordinates were converted from hg18 to hg19 using Liftover (https://genome.ucsc.edu/cgi-bin/hgLiftOver). Regions that tend to result in false-negative CNV detection, centromeric and telomeric regions (±500 kb) and immunoglobulin regions (±500 kb), were removed based on a previous study and software tutorial.

**Supporting Figures**

**S1 Fig. Overview of the study**

**S2 Fig. CNV frequency and proportion of dosage-sensitive genes overlapped by CNVs**

**S3 Fig. Enrichment of ohnologs in CNVs observed in five neuropsychiatric diseases according to the definitions of Singh, PP *et al*.**

**S4 Fig. Enrichment of dosage-sensitive genes in CNVs observed in five neuropsychiatric diseases with the use of another software, CNV Workshop**

**S5 Fig. Enrichment of ohnologs in CNVs observed in five neuropsychiatric diseases with the use of another software, CNV Workshop**

**S6 Fig. Enrichment of genes with expression sensitivity only in brain in CNVs observed in five neuropsychiatric diseases with the use of another software, CNV Workshop**

**S1 Fig. Overview of the study.** CNVs identified from each detection method were filtered and analyzed independently.

**S2 Fig. CNV frequency and proportion of dosage-sensitive genes overlapped by CNVs.** The x axis shows the frequency of CNVs in each patient and healthy individual. The y axis shows the proportion of ohnologs with SSD, singletons, ohnologs without SSD, or non-ohnologous duplicates.

*

*

*

*

*

*

*

**S3 Fig. Enrichment of ohnologs in CNVs observed in five neuropsychiatric diseases according to the definitions by Singh, PP *et al*.**

*

*

*

*

**S4 Fig. Enrichment of dosage-sensitive genes in CNVs observed in five neuropsychiatric diseases with the use of another software, CNV Workshop**

*

*

*

*

**S5 Fig. Enrichment of ohnologs in CNVs observed in five neuropsychiatric diseases with the use of another software, CNV Workshop.**

*

**S6 Fig. Enrichment of genes with expression sensitivity only in brain in CNVs observed in five neuropsychiatric diseases with the use of another software, CNV Workshop.**

**Supporting Tables**

**S1 Table. Enrichment of dosage-sensitive ohnologs in individuals with narcolepsy using CNVs detected by PennCNV**

**S2 Table. Enrichment of dosage-sensitive ohnologs in individuals with autism using CNVs detected by PennCNV**

**S3 Table. Enrichment of dosage-sensitive ohnologs in individuals with panic disorders using CNVs detected by PennCNV**

**S4 Table. Enrichment of dosage-sensitive ohnologs in individuals with essential hypersomnia using CNVs detected by PennCNV**

**S5 Table. Enrichment of dosage-sensitive ohnologs in individuals with Alzheimer disease using CNVs detected by PennCNV**

**S6 Table. Combined enrichment of dosage-sensitive genes and genes with expression sensitivity only in brain in CNVs observed in five neuropsychiatric diseases with the use of another software, CNV Workshop**

**S7 Table. Disease-specific regions for five neuropsychiatric diseases**

**S1 Table. Enrichment of dosage-sensitive ohnologs in individuals with narcolepsy using CNVs detected by PennCNV**

**S2 Table. Enrichment of dosage-sensitive ohnologs in individuals with autism using CNVs detected by PennCNV**

**S3 Table. Enrichment of dosage-sensitive ohnologs in individuals with panic disorders using CNVs detected by PennCNV**

**S4 Table. Enrichment of dosage-sensitive ohnologs in individuals with essential hypersomnia using CNVs detected by PennCNV**

**S5 Table. Enrichment of dosage-sensitive ohnologs in individuals with Alzheimer disease using CNVs detected by PennCNV**

**S6 Table. Combined enrichment of dosage-sensitive genes and genes with expression sensitivity only in brain in CNVs with the use of five neuropsychiatric diseases, with the use of other software, CNV Workshop.**

*

*

*

*

*

*

*

*

*DS: Dosage sensitive genes

*DinS: Dosage insensitive genes

*ES: genes with expression sensitivity only in brain

*-ES: rest of genes expressed genes in any tissue from GTEx.

* *: significant enrichment compared to healthy individuals.

**S7 Table. Disease-specific regions for five neuropsychiatric diseases**

References

1. Wang K, Li M, Hadley D, Liu R, Glessner J, Grant SF, et al. PennCNV: an integrated hidden Markov model designed for high-resolution copy number variation detection in whole-genome SNP genotyping data. Genome Res. 2007;17(11):1665-74.

2. Gai X, Perin JC, Murphy K, O'Hara R, D'Arcy M, Wenocur A, et al. CNV Workshop: an integrated platform for high-throughput copy number variation discovery and clinical diagnostics. BMC Bioinformatics. 2010;11:74.

3. Singh PP, Arora J, Isambert H. Identification of Ohnolog Genes Originating from Whole Genome Duplication in Early Vertebrates, Based on Synteny Comparison across Multiple Genomes. PLoS Comput Biol. 2015;11(7):e1004394.
